# Physicians' prescribing preferences were a potential instrument for patients' actual prescriptions of antidepressants^☆^

**DOI:** 10.1016/j.jclinepi.2013.06.008

**Published:** 2013-12

**Authors:** Neil M. Davies, David Gunnell, Kyla H. Thomas, Chris Metcalfe, Frank Windmeijer, Richard M. Martin

**Affiliations:** a School of Social and Community Medicine, University of Bristol, 39 Whatley Road, Bristol BS8 2PS, UK; b Medical Research Council Centre for Causal Analysis in Translational Epidemiology, School of Social and Community Medicine, University of Bristol, Bristol BS8 2BN, UK; c Department of Economics, Centre for Market and Public Organisation, University of Bristol, 2 Priory Road, Bristol BS8 1TX, UK

**Keywords:** Instrumental variables, Clinical Practice Research Datalink (CPRD), Physicians' prescribing preferences, Confounding by indication, Causality, Translational epidemiology

## Abstract

**Objectives:**

To investigate whether physicians' prescribing preferences were valid instrumental variables for the antidepressant prescriptions they issued to their patients.

**Study Design and Setting:**

We investigated whether physicians' previous prescriptions of (1) tricyclic antidepressants (TCAs) vs. selective serotonin reuptake inhibitors (SSRIs) and (2) paroxetine vs. other SSRIs were valid instruments. We investigated whether the instrumental variable assumptions are likely to hold and whether TCAs (vs. SSRIs) were associated with hospital admission for self-harm or death by suicide using both conventional and instrumental variable regressions. The setting for the study was general practices in the United Kingdom.

**Results:**

Prior prescriptions were strongly associated with actual prescriptions: physicians who previously prescribed TCAs were 14.9 percentage points (95% confidence interval [CI], 14.4, 15.4) more likely to prescribe TCAs, and those who previously prescribed paroxetine were 27.7 percentage points (95% CI, 26.7, 28.8) more likely to prescribe paroxetine, to their next patient. Physicians' previous prescriptions were less strongly associated with patients' baseline characteristics than actual prescriptions. We found no evidence that the estimated association of TCAs with self-harm/suicide using instrumental variable regression differed from conventional regression estimates (*P*-value = 0.45).

**Conclusion:**

The main instrumental variable assumptions held, suggesting that physicians' prescribing preferences are valid instruments for evaluating the short-term effects of antidepressants.

## 1 Introduction

**What is new?**

• Physicians' prior antidepressant prescribing patterns were strongly associated with their subsequent prescriptions.
• Physicians' prior antidepressant prescriptions were less strongly associated with the observable baseline characteristics of patients (potential confounders) than the actual prescriptions.
• Multiple prior prescriptions were more strongly associated with the actual prescription than a single prior prescription.
• Prior prescriptions can potentially be used to estimate treatment effects using observational data in the presence of unmeasured confounding by indication when investigating the short-term effects of antidepressants.
• There was no evidence that the association of TCAs vs. SSRIs with self-harm/death by suicide was affected by residual confounding because the results from conventional ordinary least squares regression were similar to the instrumental variable results. However, this may be because of the imprecision of the instrumental variable results.

In observational research, confounding by indication can bias estimates of drug treatment effects on outcomes that are associated with the indications for treatment [1,2]. Standard statistical approaches to deal with this adjust associations for observed covariates [3]. However, many confounders are difficult or impossible to measure [4], and a number of observational associations have been contradicted by subsequent randomized controlled trials [5,6]. This has been ascribed to unmeasured or residual confounding by indication. Observational studies can also suffer from reverse causation and protopathic biases, in which preclinical symptoms of diseases affect prescribing decisions or the ability of patients to comply with a treatment regime [7,8].

One approach to address these sources of bias is instrumental variable analysis [9–16]. This uses naturally occurring variation in likelihood of prescription, “the instrumental variable” or “instrument,” that is associated with the actual prescription but, unlike the actual prescription, is not associated with observed and unobserved confounding factors. Variation in drug prescribing associated with the instruments can provide unconfounded estimates of causal relationships between being prescribed a drug and an outcome, provided a set of assumptions are met (see Box 1).

Brookhart et al. [17] proposed that physicians' preferences for medications could be an instrumental variable for the actual prescriptions their patients received. In most observational datasets, it is not possible to directly measure physicians' prescribing preferences; therefore, Brookhart et al. argued that the prescriptions issued by physicians to their previous patients could be used as a proxy of their preferences, and hence that prior prescriptions could be used as a surrogate instrument. Brookhart et al. used this concept to estimate the effects of cyclooxygenase-2 selective inhibitors vs. traditional nonselective nonsteroidal anti-inflammatory drugs (NSAIDs) on upper gastrointestinal complications [17]. They found that physicians' prior prescriptions predicted the actual prescriptions received and that associations of potential confounders with physicians' prior prescriptions were weaker than with the actual prescriptions. Furthermore, conventional multivariate regression methods found little difference in rates of upper gastrointestinal complications by actual prescription, whereas an instrumental variable analysis, using physicians' prior prescriptions as a surrogate instrument for actual prescriptions, found evidence that patients prescribed cyclooxygenase-2 selective inhibitors had fewer upper gastrointestinal complications, in line with randomized controlled trials [18–21]. The methods have been developed in subsequent studies [15,22–29].

There has previously been concern about whether selective serotonin reuptake inhibitors (SSRIs), in particular paroxetine, cause suicide-related serious adverse events [30–33]. In this study, we evaluated physicians' prescribing preferences as an instrument for patients' prescriptions of tricyclic antidepressants (TCAs) vs. SSRIs and paroxetine vs. SSRIs, using data from the United Kingdom's Clinical Practice Research Datalink (formerly the General Practice Research Database). To evaluate the three assumptions underpinning instrumental variable analysis, we present associations of prior prescriptions (the surrogate instrument) with actual prescription and compare the strength of associations of potential confounders with prior prescriptions to that of the actual prescriptions received. We also developed the methodology by evaluating the properties of alternative instruments based on a greater number of prior prescriptions.

## 2 Methods

The Clinical Practice Research Datalink (www.cprd.com) is an administrative and clinical database containing data on over 11 million patients (4.5 million of whom are currently registered) from over 600 general practices across Britain [34]. Registered patients are representative of Britain's demography in terms of age, sex, and geographical distribution [35]. Data are validated, audited, quality checked [36,37], and have been used in over 800 peer-reviewed articles [36,38–41].

### 2.1 Study participants

We identified all patients ever registered with a practice contributing to the Clinical Practice Research Datalink before June 20, 2011, and whose records indicated that they had been prescribed an SSRI or a TCA (Appendix at www.jclinepi.com) while registered with the practice. We extracted data relating to all the antidepressant prescriptions given to these patients. We excluded prescription records if they occurred before the patients were registered at the practice, were missing a prescription date, or occurred after the patient's registration end. We kept the first prescription issued to each of the remaining patients. Of these, we excluded (1) patients first prescribed an antidepressant within 12 months of joining the practice as these may have been repeat prescriptions for medicines first prescribed by their previous general practitioner; (2) patients first prescribed antidepressants before January 1, 1995, or after June 30, 2010 (we chose 1995 because this was the first year of linked Hospital Episodes Statistics data) [42]; (3) patients issued prescriptions by staff members who were not a physician (because of ambiguity regarding who had made the prescribing decision); (4) patients' prescriptions missing the prescribing physicians' identifier; (5) patients who were younger than 10 years when first prescribed an antidepressant because prescribing in children could be for other indications; (6) patients missing the year of birth; and (7) patients whose physician previously prescribed fewer than 10 prescriptions because they had insufficient prescriptions to accurately determine preference. For the TCA vs. SSRI comparison, we excluded patients whose physician had not previously prescribed TCAs or SSRIs. For the paroxetine vs. other SSRIs comparison, we excluded patients prescribed TCAs and those whose physician had not previously prescribed an SSRI. We defined the index date as the date on which each patient received their first prescription; for the rest of the article, we refer to this prescription as the “patient's actual prescription.”

### 2.2 Actual prescription

We compared two sets of medications. First, we compared those first prescribed a TCA with those prescribed an SSRI. Second, among those prescribed SSRIs as their first antidepressant, we compared those first prescribed paroxetine with those prescribed other SSRIs.

### 2.3 Instruments

Our proposed instruments are physicians' preferences for particular antidepressants [17]. Physicians' preferences are an unobserved continuous latent variable. We used physicians' choice of antidepressant prescribed to one or more of their previous patients as a proxy for their current preferences and hence as a surrogate instrumental variable for actual prescriptions. Thus, each patient's physician was defined as having higher or lower preferences for TCAs or SSRIs among those first prescribed antidepressants and higher or lower preferences for either paroxetine or other SSRIs among those first prescribed SSRIs. Our primary surrogate instrument was the physicians' most recent prior prescription. For the TCAs vs. SSRIs comparison, we defined the surrogate instrument as equal to one if the patient's physician previously prescribed a TCA and zero if they prescribed a SSRI. For the paroxetine vs. nonparoxetine SSRI analysis, we defined the surrogate instrument as equal to one if the physician previously prescribed paroxetine and zero if they prescribed a nonparoxetine SSRI.

**Fig. 1:**
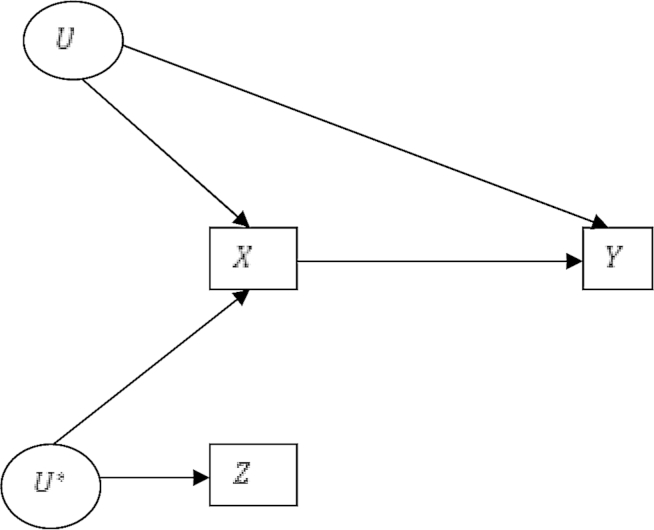
Directed acyclic graph of outcomes *Y*, prescriptions *X*, instrumental variable the unmeasured physician prescribing preference U*, surrogate instrument physicians' prior prescriptions *Z*, and unmeasured confounders *U*.

The assumptions defining an instrumental variable are described in Box 1 and illustrated in Fig. 1.

Physicians' preferences are likely to affect many of their previous prescriptions, not just their most recent prior prescription. Therefore, we hypothesized that alternative measures of prior prescriptions may be more strongly associated with each patient's actual prescription. In a secondary analysis, we also investigated whether we could construct better instruments using multiple prior prescriptions from each physician. Specifically, we investigated whether two pragmatically defined alternative specifications of surrogate instruments, using longer prescription histories for each physician, could increase the strength of the association of the prior prescriptions with the actual prescriptions. First, we used a count of the number of TCA (or paroxetine) prescriptions issued by each physician to their previous three patients. Second, we defined a set of indicator variables for each physician's previous seven prescriptions. We defined these alternative instruments without first looking at the data as defining instruments because of the strength of their association with the exposure can lead to bias [43–45].

### 2.4 Potential confounding factors

We investigated associations of our surrogate instruments with the following potential confounding factors (factors associated with both suicide risk and prescribing decisions [46]): body mass index (BMI), hospitalization in the prior year, having more than 13 primary care consultations in the prior year, age, having more than five prescriptions in the prior year, gender, ever smoked, prior diagnosis of depression or self-harm, previous prescription of hypnotics (British National Formulary chapter 4.1.1) or antipsychotics (British National Formulary chapter 4.2.1 or 4.2.2), whether the patient had a nonzero Charlson Index (an index of morbidity) before prescription, and year of first prescription (to account for prescribing trends) [47]. We excluded records with a BMI of more than 100 or less than 15 in case they were data errors.

### 2.5 Tests of the instrumental variable assumptions

We investigated the validity of the first assumption [A], by testing the strength of association of the prior prescriptions with the actual prescription using a partial *F*-test [11,48] from a regression of the actual prescription on prior prescription adjusted for prescription year, using robust standard errors clustered by the physician who issued the prescription [49]. The partial *F*-statistic tests whether the instruments are associated with the exposure after conditioning on included covariates (in this case year of first prescription).

We evaluated whether assumption [C] held by estimating risk differences of each confounding factor by patients' actual prescriptions and by measures of the prior prescriptions. We estimated these risk differences for patients first prescribed TCAs vs. those prescribed SSRIs and for those prescribed paroxetine vs. those prescribed nonparoxetine SSRIs, using crude and multivariate-adjusted ordinary least squares regression, with robust standard errors clustered by physician [49]. We used ordinary least squares rather than logistic regression to allow comparison of our results with previous articles [17,23]. Prescription rates for each drug changed over time so all models control for the year of first prescription. We also report the Mahalanobis distance, a summary measure of the total covariate imbalance, for both prior prescriptions (the instrument) [23,50] and actual prescriptions. We report the percentage reduction in the Mahalanobis distance between the actual prescriptions and the instruments.

We estimated the prevalence difference ratio to assess whether the bias due to each potential confounding factor was likely to be larger in a conventional or instrumental variables analysis [24]:

$$\text{pdr}=\frac{E\left(U|Z=1\right)-E\left(U|Z=0\right)}{E\left(U|X=1\right)-E\left(U|X=0\right)}$$

where *U* indicates the covariate of interest, and *Z* and *X* are the prior and actual prescriptions, respectively. If the prevalence difference ratio is greater than the strength of the surrogate instrument E(X|Z=1)−E(X|Z=0), then the bias due to the confounder in the instrumental variable results may be greater than the conventional results. So, if physicians who previously prescribed a TCA prescribed TCAs to 15 extra patients per 100 and the prevalence difference ratio is greater than 0.15, then the instrumental variable results may be more biased than the conventional ordinary least squares results.

We investigated the associations of the surrogate instruments based on longer treatment histories (count of the previous three and indicator variables for the seven previous prescriptions) with the actual prescriptions and the observed covariates. We regressed each covariate on the set of surrogate instruments and adjusted for year of prescription. We used *F*-tests to assess the joint null hypothesis of no association of the prior prescriptions with the covariates. We estimated the strength of associations of each definition of surrogate instruments and actual prescription. We tested whether the additional prescriptions explained any additional variance in the exposure using a Lagrange multiplier test [51].

We used these instruments to investigate whether patients prescribed TCAs were more likely to die by suicide or be admitted to hospital for self-harm compared with those prescribed SSRIs using linked administrative data from the Office of National Statistics (for mortality data) and Hospital Episode Statistics (for admission data). These outcomes have been previously validated [52]. We only had linked data for 50% of general practices, so we limited this analysis to these practices. We tested for differences in the effect estimates between conventional ordinary least squares regression and instrumental variable regression using a Durbin–Wu–Hausman test [53,54], and compared these results to a propensity score–adjusted analysis. We derived propensity scores using sampling with replacement and a tolerance in the difference in the propensity score between matched patients of 0.01. We included all the covariates listed in Table 1 except BMI because BMI had missing values. We undertook three sensitivity analyses. First, we tested whether there was any evidence of a direct effect of physicians on suicide or self-harm by including physician fixed effects (using an indicator variable for each of the 3,042 physicians in the sample). Second, we repeated the analysis excluding low-dose amitriptyline (i.e., prescriptions <13 mg in one analysis and <70 mg in another) because such doses may have been prescribed for nondepression diagnoses such as neuropathic pain. Finally, we increased the precision of the results using seven previous prescriptions.

All analyses were conducted in Stata (version 12.0 StataCorp, TX), using robust standard errors clustered by physician; we used the command IVREG2 for the instrumental variable analyses, XTIVREG2 for the fixed-effects instrumental variable analyses, and propensity scores were derived using PSMATCH2 [55–57].

## 3 Results

We identified 897,983 patients prescribed either SSRIs or TCAs between January 1, 1995, and June 30, 2010. We dropped 11,248 patients whose physician issued fewer than 10 prescriptions or had not previously issued a prescription. This left 886,735 patients, who attended 6,555 physicians at 612 general practices, for inclusion in the analysis. Altogether, 484,858 patients were issued a TCA and 401,877 an SSRI, 45,238 of whom were issued paroxetine. On average, each physician issued 135 in total, or 21 per year, first-time antidepressant prescriptions. In the 3 months after first prescription, we identified 608 cases of death by suicide or hospital admission for self-harm, an incident rate of 0.15 per 100 patients treated, or 600 per 100,000 patient-years.

We found evidence of differences in baseline variables (potential confounders) between patients prescribed TCAs and those prescribed SSRIs (Table 1). The only covariate in which there was no difference was a prior diagnosis of self-harm. The differences between the actual prescription groups were considerable: those prescribed TCAs (vs. SSRIs) were 21.3 percentage points more likely to be more than 40 years of age, 22.5 percentage points more likely to have had more than five prescriptions of any type in the year before the antidepressant was issued, and were 8.8 percentage points more likely to have a Charlson index of at least one. Although we found evidence of differences between patients depending on their physicians' previous prescription (the instrument), these differences were considerably smaller than when comparing what was actually prescribed: patients whose physician previously prescribed a TCA were 2.5 percentage points more likely to be more than 40 years of age, 2.1 percentage points more likely to have been issued more than five prescriptions in the previous year, and 1.2 percentage points more likely to have a Charlson index of at least one. Patients prescribed TCAs were 5.0 percentage points more likely to be prescribed before 2004, the risk difference was similar for prior prescriptions, therefore prior prescriptions are unlikely to reduce confounding by prescription year. The total imbalance in the covariates, as measured by the Mahalanobis distance, was 83% lower for the prior prescription than the actual prescription.

We found smaller differences between those issued prescriptions for paroxetine vs. those issued other SSRIs (Table 2): patients prescribed SSRIs were 2.2 percentage points less likely to have had more than 13 consultations in the year before the prescription, 4.0 percentage points more likely to be male, and 5.2 percentage points less likely to be diagnosed with depression before the index prescription. There was little evidence of differences by the prior type of SSRI prescription for most patient characteristics, except BMI and gender (patients whose physician previously prescribed paroxetine were 0.56 percentage points more likely to have BMI more than 25 and 0.51 percentage points more likely to be male). However, the type of prescription prescribed changed over time, probably reflecting concerns about suicide risk associated with paroxetine [58]. Thus, patients whose physician previously prescribed paroxetine were 49.4 percentage points more likely to have received their prescription before 2004; therefore, we adjusted all our results for year of first prescription. The Mahalanobis distance was 22% lower for prior prescriptions of paroxetine vs. other SSRIs than for actual prescriptions. This difference is considerably smaller than the 83% reduction seen in the TCAs vs. SSRIs (see above).

We found that physicians' previous prescriptions were strongly associated with patients' actual prescriptions (Table 3). Physicians who previously prescribed a TCA were 15 percentage points more likely to prescribe a TCA to their next patient, and physicians who previously prescribed paroxetine were 28 percentage points more likely to prescribe paroxetine to their next patient. The partial *F*-test was 3,663 for TCAs vs. SSRIs and 2,770 for SSRIs vs. paroxetine, suggesting that prior prescriptions are sufficiently strong to use as surrogate instruments for actual prescriptions.

The prevalence difference ratios (Table 4) suggest that instrumental variable–based results for TCAs (vs. SSRIs) have smaller bias (prevalence difference ratio <14.95, Table 3) than that of the conventional analysis, for all covariates except hospitalization in the prior year, gender, or a prior diagnosis of self-harm. For these variables, the strength of the association of confounder with actual prescription was relatively weak, implying that the bias due to these variables may be relatively small. The prescribing difference ratios for paroxetine suggest that for only 6 of the 12 covariates would the instrumental variable results have smaller bias (prevalence difference ratio smaller than 28% for number of consultations, number of prescriptions, gender, age at first prescription, a prior diagnosis of depression, prior prescription of an antipsychotic). This may be because the strength of the associations of actual prescriptions with the covariates was weaker for SSRIs vs. paroxetine than for TCAs compared with SSRIs.

Including more prior prescriptions in the definition of the surrogate instrument increased the strength of the joint association with actual prescription (Appendix at www.jclinepi.com). For TCAs, the *F*-test was 3,663 for one prior prescription compared with 1,971 for the count of the previous three prescriptions. Including indicator variables for the physicians' seven previous prescriptions increased the strength of the association further; the *F*-test was 1,469, which reduced the standard error to 0.09. In addition to the variance in exposure explained by a single prior prescription, the six extra prescriptions explained more of the variance in the exposure (Langrage multiplier test = 1,622 ∼ *χ*^2^ [6]; *P* < 0.001). Each of the prior prescriptions, when included individually, was independently associated with actual prescription. Similarly for paroxetine, the count of the previous prescriptions was more strongly associated with the actual prescription than a single prior prescription (Appendix at www.jclinepi.com) (Langrage multiplier test = 925 ∼ *χ*^2^ [6]; *P* < 0.001). Including extra prescriptions reduced the sample by 49,330 (4.4%) for the TCAs vs. SSRIs analysis and by 30,864 (7.9%) for the paroxetine vs. other SSRIs analysis (because the first seven patients seen by each physician have fewer than seven prior prescriptions to include in the analysis).

There was little evidence that including more prior prescriptions in the definition of the surrogate instrument increased the association with the confounders (Appendix at www.jclinepi.com). For TCAs vs. SSRIs, both the count of three prior prescriptions and indicator variables for the seven prior prescriptions had a smaller *F*-statistic than the single prior prescription. However, neither definition using multiple instruments consistently had the lowest association with the covariates. For paroxetine vs. SSRIs, the count of three prior prescriptions was more strongly associated with the confounders than a single prior prescription (Appendix at www.jclinepi.com), but there was little evidence that the seven prior prescriptions were jointly associated with any of the covariates.

Using conventional ordinary least squares regression, we found that fewer patients prescribed TCAs had an admission to hospital for self-harm or died by suicide than those prescribed SSRIs (risk difference per 100 patients prescribed [RD], −0.11; 95% confidence interval [CI], −0.14, −0.08; Table 5). In contrast, the risk difference calculated using one prior prescription as the instrumental variable was attenuated by approximately 50% toward the null (RD = −0.04; 95% CI, −0.21, 0.13), although the CI was wider (less precise) and we could not reject the null hypothesis of no difference between the conventional and instrumental variable analyses (Hausman test: *P* = 0.45). The results based on seven prior prescriptions were more precise (RD = −0.10; 95% CI, −0.20, 0.01) but remained consistent with the conventional analysis. The results using physician fixed effects were similar to those based on one prior prescription as the instrument. Excluding low-dose amitriptyline (either <13 or <70 mg) made no meaningful differences to the results. The propensity score–adjusted results indicated a similar magnitude of effect but were more precise than the instrumental variable results.

## 4 Discussion

Physicians' previous prescriptions of antidepressants were associated with their patients' actual prescriptions of antidepressants. This is consistent with the findings of previous studies of other medications that found that physicians' previous prescriptions can be used as surrogate instruments for prescriptions of NSAIDs and antipsychotics [17,27]. We found that including more prior prescriptions increased the strength of the association between prior prescriptions and actual prescription. The patients' actual prescriptions were strongly associated with patients' characteristics and prescription histories. This suggests that confounding bias is possible in conventional estimates using multivariate adjustment or a propensity score. If observed covariates are correlated with the outcome and exposure, then unobserved (and unmeasured) confounding variables may also be correlated with outcome and the exposure. In contrast, the prior prescriptions were less strongly associated with the observed covariates. There was little evidence that the strength of these associations with covariates increased when we used a greater number of prior prescriptions as surrogate instruments. For many of the covariates, particularly the covariates most strongly associated with the actual prescriptions such as prior diagnosis of depression and number of prior prescriptions, instrumental variable estimates using prior prescriptions may have smaller bias than conventional analysis, as can be seen in Table 4 [24].

Although the instrumental variable estimate of the effect of TCAs vs. SSRIs on self-harm and death by suicide was attenuated by approximately 50% toward the null compared with the results from conventional regression, the CIs of the instrumental variable estimates were wide, and there was little statistical evidence of any difference between the conventional and instrumental variable analysis (Hausman test: *P* = 0.45). This implies that the conventional results may not suffer from residual or uncontrolled confounding, although our sample size is likely to have been underpowered to detect differences between the conventional and instrumental variable estimates. The propensity score results were attenuated compared with the conventional regression results but were consistent with the instrumental variable results. The instrumental variable results (based on one prior prescription) suggested that prescribing TCAs (rather than SSRIs) was unlikely to reduce risk by more than 0.21 or increase risk by more than 0.13 events per 100 patients treated. This compares with a suicide or self-harm incidence rate of 0.15 per 100 patients treated, suggesting that prescribing TCAs is unlikely to more than double the risks of suicide or self-harm compared with SSRIs. The instrumental variable results based on seven prior prescriptions were more precise, suggesting TCAs were unlikely to reduce risk by more than 0.20 or increase risk by more than 0.01 per 100 patients treated compared with SSRIs.

Instrumental variable results using prior prescriptions estimate the effects of being prescribed paroxetine or TCAs relative to being prescribed other SSRIs. The specific patients for whom the effects of prescription would be identified depend on the assumptions used to identify the results. If the results are identified by assuming a monotonic effect of physicians' preferences on likelihood of prescription (e.g., that a patient prescribed paroxetine by a particular physician would also have been prescribed paroxetine in a counterfactual situation in which they attended a physician with higher preferences for paroxetine), the results would reflect an average effect of prescription in the group of patients affected by their physicians' preferences. An alternative identifying assumption is no effect modification by values of the instrument. This assumes that the physicians' previous prescriptions do not change the effect of the medication. If the results are identified by no effect modification, then we would identify the effects of prescription on those prescribed. Both the assumption of no effect modification and monotonicity are metaphysical assumptions; therefore, there is no way to test if they hold [59]. However, because the instruments do not necessarily estimate an average treatment effect across the entire population, they may not be comparable with results from a randomized controlled trial. The instrumental variable results also may not reflect the treatment effects in patients who are never likely to be prescribed one of the drugs, for example, those with strong contraindications (e.g., paroxetine in children).

Although we cannot assess the degree of confounding by unobserved variables, the relative strength of associations of several observed characteristics, with first, the surrogate instrument (prior prescriptions) and second, the patient's actual prescription, gives an indication of the likely associations with potential unobserved confounding variables [59].

Three previous studies have investigated the properties of different definitions of physicians' prescribing preferences. Hennessey et al. [60] found that the physicians' most recent prior prescriptions of NSAIDs were more strongly associated with their patients' actual prescriptions than older prescriptions. Ionescu-Ittu et al. [61] found that the instrument most strongly associated with patients' treatments was the proportion of each hospital's previous patients who were treated with rhythm vs. rate control therapy for atrial fibrillation. Finally, Rassen et al. [23] found that restricting their analysis to high-volume practices maximized the strength of their instrument for prescribing antipsychotic medications, and consequently the precision of their results. We add to these findings in three ways. First, physicians' prior prescriptions are potentially valid surrogate instruments for antidepressants in the United Kingdom's Clinical Practice Research Datalink as they were associated with physicians' subsequent prescriptions and less associated with potential confounders than patients' actual prescriptions. Second, using multiple prior prescriptions as the instrument increased the precision of the instrumental variable estimates. Finally, we specified the assumptions needed to estimate the effects of antidepressants on an outcome and described the patients for whom we could identify the effects of prescription.

### 4.1 Strengths and limitations

Our sample is the largest ever used in an investigation of physicians' prescribing preferences and the first study to investigate alternative definitions of surrogate instruments using multiple prior prescriptions in the Clinical Practice Research Datalink. The prescribing data are of high quality and measure prescriptions that the physicians issued to their patients. This is also the first study to investigate whether physicians' preferences are valid instruments for prescriptions of antidepressants. As we used data from standard clinical care, rather than data collected specifically to evaluate a particular drug, the results may be more representative of the effects of prescriptions as used in the community.

A limitation of our study is that we only have data on prescriptions issued, not on prescriptions cashed. This means we cannot identify the effects of treatment, only the effects of being issued a prescription. Hence, if a medication has poor compliance when used in mainstream clinical care, then instrumental variable results may be attenuated relative to the results of a randomized controlled trial with greater compliance. However, this intention-to-treat parameter may be of more interest to physicians who have less influence over compliance than they do over which medication is issued. Therefore, we do not believe this to be a major limitation. Another limitation was that we only investigated data from the United Kingdom, so these findings may not necessarily hold in other settings. But, our findings are consistent with previous international studies, which suggests physicians' prescribing preferences may be more widely valid. Finally, our results investigating the validity and utility of physicians' prescribing preferences for suicide and self-harm are not necessarily informative for other outcomes.

## 5 Conclusions

Physicians' previous prescriptions are potential surrogate instruments for patients' actual prescriptions when investigating the effects of antidepressants and can address problems of unmeasured and residual confounding in observational studies investigating the effects of antidepressants. We found that prior prescriptions of antidepressants were less associated with observed confounders; hence, they may be less associated with unobserved confounders as well. There was more evidence of associations between covariates and actual prescription in the TCA vs. SSRI analysis, suggesting that analyses comparing TCAs with SSRIs may be more likely to suffer from unmeasured or residual confounding than when comparing paroxetine to other SSRIs. We demonstrated that including more than one previous prescription as surrogate instruments for each physician's preferences can potentially increase the precision of instrumental variable results. The most precise specification used seven previous prescriptions. Using conventional regression, we found evidence that patients prescribed TCAs were less likely to be admitted to hospital for self-harm or die by suicide. Our instrumental variable regressions were less precise and found little evidence of a large difference in suicide or self-harm between TCAs and SSRIs. Despite their imprecision, our instrumental variable results based on one prior prescription can exclude a more than doubling of risk of suicide or self-harm among those prescribed TCAs vs. SSRIs. Our instrumental variable results based on seven prior prescriptions were precise enough to exclude an increased risk of suicide or self-harm among those prescribed TCAs vs. SSRIs and were consistent with the conventional results. The continued development of large administrative databases, such as the Clinical Research Practice Datalink, provides an ideal source of data for instrumental variable analysis.

## Figures and Tables

(data from Hernán and Robins [15])

**Table 1:** Potential confounders by first antidepressant prescribed (TCAs vs. SSRIs)

Variable	Actual prescription		Physicians' prior prescription		Risk difference per 100, TCAs vs. SSRIs	
	TCAs (%)	SSRIs (%)	TCAs (%)	SSRIs (%)	Actual prescription	Physicians' prior prescription
*N*	484,858	401,877	484,692	402,043		
BMI > 25 kg/m^2^ (*N* = 679,755)	57.7	50.7	55.0	54.1	7.08^b^	0.92^b^
Hospitalized in prior year	0.4	0.3	0.4	0.4	0.10	−0.02
More than 13 consultations in prior year	76.8	58.6	69.2	67.7	18.42	1.50
Older than 40 at first prescriptions	71.9	50.8	63.5	61.0	21.28	2.50
More than five prescriptions in prior year	71.1	48.9	62.0	59.9	22.45	2.13
Male^a^	37.7	39.6	38.8	38.3	−1.83	0.46
Ever smoked	40.9	56.8	47.2	49.3	−16.11	−2.15
Diagnosed depressed before prescription	43.2	62.0	50.6	53.1	−19.01	−2.56
Prior diagnosis of definite self-harm	5.8	5.8	5.9	5.7	−0.04	0.16
Prior hypnotic prescriptions	16.5	12.8	14.6	15.1	3.78	−0.59
Prior antipsychotic prescriptions	2.4	2.0	2.3	2.2	0.42	0.05
Prior Charlson Index not zero	42.1	33.4	38.7	37.5	8.83	1.18
Percent prescribed before 2004	50.8	45.9	50.8	45.8	4.95	5.04
Mahalanobis distance	18.5	16.8	17.9	17.6	1.75	0.29
Reduction in Mahalanobis distance						−83%

*Abbreviations*: TCA, tricyclic antidepressant; SSRI, selective serotonin reuptake inhibitor; BMI, body mass index. All estimates adjusted for year of first prescription. The number of patients in this table is more than those prescribed SSRIs in Table 2 because of 11,277 patients whose physicians had previously prescribed at least 10 TCAs but prescribed fewer than 10 SSRIs.

a One patient missing gender.

b Mean difference.

**Table 2:** Potential confounders by first antidepressant prescribed (paroxetine vs. nonparoxetine SSRIs)

Variable	Actual prescription		Physicians' prior prescription		Risk difference per 100 paroxetine vs. other SSRIs	
	Paroxetine (%)	SSRIs (%)	Paroxetine (%)	SSRIs (%)	Actual prescription	Physicians' prior prescription
*N*	44,470	346,130	45,238	345,362		
BMI > 25 kg/m^2^ (*N* = 290,301)	49.1	49.9	50.3	49.9	−0.91	0.56
Hospitalized in prior year	0.3	0.3	0.4	0.3	0.02	0.10
More than 13 consultations in prior year	57.5	59.4	59.0	59.2	−2.23	−0.26
Older than 40 at first prescriptions	50.0	49.7	49.9	49.8	0.34	0.06
More than five prescriptions in prior year	47.7	49.4	49.1	49.2	−1.97	−0.15
Male^a^	42.6	39.2	40.0	39.6	3.99	0.51
Ever smoked	56.7	56.4	56.6	56.4	0.37	0.25
Diagnosed depressed before prescription	56.7	61.1	60.4	60.8	−5.18	−0.45
Prior diagnosis definite self-harm	6.4	6.2	6.4	6.2	0.26	0.23
Prior hypnotic prescriptions	13.0	12.8	12.6	12.8	0.18	−0.22
Prior antipsychotic prescriptions	2.5	1.9	2.0	2.0	0.69	0.01
Prior Charlson Index not zero	32.7	33.8	34.1	33.7	−1.31	0.43
Percent prescribed before 2004	89.6	40.2	89.5	40.1	49.43	49.36
Mahalanobis distance	17.9	16.8	17.7	16.9	1.11	0.87
Reduction in Mahalanobis distance						−22%

*Abbreviations*: SSRI, selective serotonin reuptake inhibitors; BMI, body mass index. All statistics adjusted for year of first prescription. Definition of SSRI excludes paroxetine. The number of patients in this table is fewer than those prescribed SSRI in Table 1 because of 11,277 patients whose physicians had previously prescribed at least 10 antidepressants but prescribed fewer than 10 SSRIs. ^b^ Mean difference.

a One patient missing gender.

**Table 3:** Association of patients' actual prescription with physicians' previous prescription for TCAs vs. SSRIs and paroxetine vs. SSRIs, adjusted for year of first prescription

Instrument	TCAs vs. SSRIs	Paroxetine vs. SSRIs
	Risk difference (95% CI)	Risk difference (95% confidence interval)
Prior prescription	14.90 (14.42, 15.38)	27.72 (26.69, 28.76)
*N*	886,735	390,600
Number of physicians (clusters)	6,555	5,144
*F*-test	*F*(1, 6554) = 3,663	*F*(1, 5143) = 2,770
Partial *r*^2^	0.02	0.08

*Abbreviations*: TCA, tricyclic antidepressant; SSRI, selective serotonin reuptake inhibitor; CI, confidence interval. All CIs robust for heteroskedasticity and clustered by physician. Risk difference is difference in probability of TCA or paroxetine actually being prescribed if the physician previously prescribed a TCA or paroxetine (e.g., in row 1, if the physician previously prescribed a TCA, their current patient is 15% more likely to also be prescribed a TCA than a SSRI).

**Table 4:** Prevalence difference ratios for TCAs vs. SSRIs and paroxetine vs. SSRIs

Variable	Prevalence difference ratio	
	TCAs vs. SSRIS (%)	Paroxetine vs. SSRIs (%)
*N*	886,735	390,600
BMI > 25 kg/m^2^ (*N* = 290,756)	13.3	−61.8
Hospitalized in prior year	−19.9	493.1
More than 13 consultations in prior year	8.4	11.8
Older than 40 at first prescriptions	11.8	18.9
More than five prescriptions in prior year	9.7	7.6
Male	−28.6	12.9
Ever smoked	13.5	68.0
Diagnosed depressed before prescription	13.4	8.8
Prior diagnosis definite self-harm	−292.7	89.2
Prior hypnotic prescriptions	−14.6	−121.2
Prior antipsychotic prescriptions	12.2	0.9
Prior Charlson Index not zero	14.5	−32.9

*Abbreviations*: TCA, tricyclic antidepressant; SSRI, selective serotonin reuptake inhibitor; BMI, body mass index. When the prevalence difference ratio is greater than the strength of the instrument's association with the actual prescriptions, then the instrumental variable results may be more biased than the conventional results. The strength of the association for the TCAs vs. SSRIs analysis was 15% and for paroxetine vs. SSRIs it was 28%.

**Table 5:** Conventional multivariate regression, instrumental variable, and propensity score estimates of risk differences of hospital admission for self-harm (Hospital Episode Statistics data) or death by suicide (Office of National Statistics mortality data) within 3 months of index prescription (number of patients = 394,846^a^ and number of physicians = 3,042)

	TCAs vs. SSRIs risk differences (95% CI)				
	Ordinary least squares regression	Instrumental variable analysis using one prior prescription	Instrumental variable analysis using one prior prescription and physician fixed effects	Instrumental variable analysis using seven prior prescriptions	Propensity score adjustment^b^
TCA (reference category SSRIs)	−0.11 (−0.14, −0.08)	−0.04 (−0.21, 0.13)	−0.06 (−0.58, 0.45)	−0.10 (−0.20, 0.01)	−0.05 (−0.08, −0.03)
*F*-test		1,727	596		
Hausman test (*P*-value)		0.45	0.83		

*Abbreviations*: TCA, tricyclic antidepressant; SSRI, selective serotonin reuptake inhibitor; CI, confidence interval; BMI, body mass index. CIs allow for clustering by physician. Patients previously admitted to hospital for self-harm are omitted. Reported *F*-statistic is robust and is a test of the partial association of the instrument and the prescription. The null hypothesis of the Hausman test is that there is no difference between the conventional ordinary least squares estimates and the instrumental variable results.

a Based on the 50% of practices that were linked to the Hospital Episodes Statistics and ONS databases.

b *N* = 394,836 because one patient with missing gender was excluded and nine patients were not in the common support for the propensity score adjustment results. Propensity score based on all covariates in Table 1 except BMI because BMI had 86,488 missing values.

## Box 1 The assumptions for instrumental variable analysis and how they are met in this study

**Table:**

Assumption	How assumptions are met	
	True instrument (preference)	Surrogate instrument (prior prescription)
[A] The instrumental variable is associated with the actual prescription.	Physicians' preferences for particular antidepressants are associated with the actual prescription they issue to their current patient.	A physician's prescription to their previous patient is a surrogate or proxy for their preferences. This surrogate is associated with the actual prescription they issue to their next patient.
[B] The instrumental variable does not directly affect the outcomes.	Physicians' preferences for particular antidepressants are unlikely to directly affect their patients' outcomes.	A physician's prescription to their previous patient is unlikely to directly affect their next patient's outcomes.
[C] The instrumental variable is not associated with the confounding factors.	Physicians' preferences for particular antidepressants may not be associated with potential confounding factors (e.g., an individual with a history of self-harm) because patients' choices of physician are quasi-random.	A physician's prescription to their previous patient may not be associated with their next patient's characteristics.
