# Supplementary material for: Physicians' prescribing preferences were a potential instrument for patients' actual prescriptions of antidepressants
Source: J Clin Epidemiol. 2013 Dec;66(12):1386–96. doi: 10.1016/j.jclinepi.2013.06.008 (PMC3824069; doi:10.1016/j.jclinepi.2013.06.008)
Supplement: Supplementary Tables [file mmc1.pdf]

eTable 1: Codes used to defined tricyclic antidepressants.

multilexcode	Product Name
04425002	perphenazine with amitriptyline tablets 2mg + 25mg
04425001	perphenazine with amitriptyline tablets 2mg + 10mg
05296001	amitriptyline hydrochloride with perphenazine tablets 10mg + 2mg
00982002	TRIPTAFEN M tablets 2mg + 10mg [GOLDSHIELD]
05296002	amitriptyline hydrochloride with perphenazine tablets 25mg + 2mg
00982001	TRIPTAFEN tablets 2mg + 25mg [GOLDSHIELD]
16379001	TRIPTAFEN M tablets 2mg + 10mg [GOLDSHIELD]
01870001	TRYPTIZOL injection 10mg/ml [M S D]
03075001	amitriptyline hydrochloride injection 10mg/ml
05295002	amitriptyline hydrochloride with chlordiazepoxide capsules 25mg + 10mg
00527001	LIMBITROL 5 capsules [ROCHE]
05295001	amitriptyline hydrochloride with chlordiazepoxide capsules 12.5mg + 5mg
01656001	LIMBITROL 10 capsules [ROCHE]
05994003	amoxapine tablets 100mg
05994002	amoxapine tablets 50mg
05990002	ASENDIS tablets 50mg [WYETH PHAR]
05995001	amoxapine tablets 150mg
05990001	ASENDIS tablets 25mg [WYETH PHAR]
05991001	ASENDIS tablets 150mg [WYETH PHAR]
05990003	ASENDIS tablets 100mg [WYETH PHAR]
05994001	amoxapine tablets 25mg
05311001	butriptyline tablets 25mg
01865001	EVADYNE tablets 25mg [WYETH PHAR]
05311002	butriptyline tablets 50mg
01855003	ANAFRANIL capsules 50mg [NOVARTIS]
00702009	CLOMIPRAMINE capsules 10mg [HILLCROSS]
03360001	clomipramine syrup 25mg/5ml
02451009	CLOMIPRAMINE capsules 10mg [IVAX]
02451010	CLOMIPRAMINE capsules 25mg [IVAX]

eTable 1: Codes used to defined tricyclic antidepressants.

multilexcode	Product Name
16121001	clomipramine oral suspension 50mg/5ml
03360002	clomipramine injection 12.5mg/ml
01855002	ANAFRANIL capsules 25mg [NOVARTIS]
01855001	ANAFRANIL capsules 10mg [NOVARTIS]
01659009	CLOMIPRAMINE capsules 10mg [TEVA]
03359001	clomipramine capsules 10mg
01857001	ANAFRANIL SR tablets 75mg [NOVARTIS]
01856001	ANAFRANIL syrup 25mg/5ml [NOVARTIS]
03362001	ANAFRANIL injection 12.5mg/ml [NOV/GEIGY]
01659010	CLOMIPRAMINE capsules 25mg [TEVA]
03359002	clomipramine capsules 25mg
00702011	CLOMIPRAMINE capsules 50mg [HILLCROSS]
00702010	CLOMIPRAMINE capsules 25mg [HILLCROSS]
03361001	clomipramine modified release tablet 75mg
02451011	CLOMIPRAMINE capsules 50mg [IVAX]
03359003	clomipramine capsules 50mg
11093002	THADEN tablets 75mg [OPUS]
02237010	DOSULEPIN tablets 75mg [ACTAVIS]
03131009	DOSULEPIN capsules 25mg [GEN (UK)]
01648010	DOSULEPIN capsules 25mg [TEVA]
01648009	DOSULEPIN tablets 75mg [TEVA]
03688003	dosulepin sugar free elixir 25mg/5ml
02237009	DOSULEPIN capsules 25mg [ACTAVIS]
00385010	DOSULEPIN tablets 75mg [HILLCROSS]
11093001	THADEN capsules 25mg [OPUS]
03841009	DOSULEPIN capsules 25mg [KENT]
02277001	PREPADINE capsules 25mg [BERK]
02277002	PREPADINE tablets 75mg [BERK]
01873001	PROTHIADEN capsules 25mg [TEOFARMA]
17359001	dosulepin sugar free oral solution 100mg/5ml

eTable 1: Codes used to defined tricyclic antidepressants.

multilexcode	Product Name
01672001	dosulepin sugar free elixir 75mg/5ml
01436010	DOSULEPIN capsules 25mg [IVAX]
00385009	DOSULEPIN capsules 25mg [HILLCROSS]
04751009	DOSULEPIN capsules 25mg [SANDOZ]
02181002	DOTHAPAX tablets 75mg [ASHBOURNE]
01873002	PROTHIADEN tablets 75mg [TEOFARMA]
05198009	DOSULEPIN capsules 25mg [ALMUS]
03532009	DOSULEPIN capsules 25mg [SOVEREIGN]
03688002	dosulepin tablets 75mg
02181001	DOTHAPAX capsules 25mg [ASHBOURNE]
01436009	DOSULEPIN tablets 75mg [IVAX]
05199009	DOSULEPIN tablets 75mg [ALMUS]
03688001	dosulepin capsules 25mg
01672002	dosulepin mixture 25mg/5ml
03131010	DOSULEPIN tablets 75mg [GEN (UK)]
01876001	SINEQUAN capsules 75mg [PFIZER]
01875001	SINEQUAN capsules 10mg [PFIZER]
03692001	doxepin capsules 75mg
01875002	SINEQUAN capsules 25mg [PFIZER]
14828001	SINEPIN capsules 50mg [MARLBORO]
03691003	doxepin capsules 50mg
01875003	SINEQUAN capsules 50mg [PFIZER]
03691001	doxepin capsules 10mg
03691002	doxepin capsules 25mg
14827001	SINEPIN capsules 25mg [MARLBORO]
16793001	doxepin oral suspension 25mg/5ml
02365001	MOTIVAL tablets [SANOFI S]
02367001	MOTIPRESS tablets 30mg + 1.5mg [SANOFI S]
05369002	nortriptyline with fluphenazine tablets 30mg + 1.5mg
03500001	fluphenazine with nortriptyline tablets 500mcg + 10mg

eTable 1: Codes used to defined tricyclic antidepressants.

multilexcode	Product Name
05369001	nortriptyline with fluphenazine tablets 10mg + 500micrograms
03890002	iprindole hydrochloride tablets 30mg
03890001	iprindole hydrochloride tablets 15mg
03891002	PRONDOL tablets 30mg [WYETH PHAR]
03891001	PRONDOL tablets 15mg [WYETH PHAR]
10794001	LOMONT sugar-free suspension 70mg/5ml [ROSEMONT]
02256009	LOFEPRAMINE tablets 70mg [ACTAVIS]
01922009	LOFEPRAMINE sugar-free suspension 70mg/5ml [ROSEMONT]
02807009	LOFEPRAMINE tablets 70mg [HILLCROSS]
03036009	LOFEPRAMINE tablets 70mg [STERWIN]
02138009	LOFEPRAMINE tablets 70mg [IVAX]
04000001	lofepramine tablets 70mg
02857009	LOFEPRAMINE tablets 70mg [TEVA]
04000002	lofepramine sugar-free suspension 70mg/5ml
01867001	GAMANIL tablets 70mg [MERCK]
01851001	LUDIOMIL tablets 10mg [NOV/CIBA]
01851003	LUDIOMIL tablets 50mg [NOV/CIBA]
04072001	maprotiline tablets 75mg
04071002	maprotiline tablets 25mg
01851002	LUDIOMIL tablets 25mg [NOV/CIBA]
04071003	maprotiline tablets 50mg
04071001	maprotiline tablets 10mg
01852001	LUDIOMIL tablets 75mg [NOV/CIBA]
00117003	BOLVIDON tablets 30mg [ORGANON]
04190003	mianserin tablets 30mg
04190002	mianserin tablets 20mg
00117001	BOLVIDON tablets 10mg [ORGANON]
00661002	NORVAL tablets 20mg [BENCARD]
00117002	BOLVIDON tablets 20mg [ORGANON]
04190001	mianserin tablets 10mg

eTable 1: Codes used to defined tricyclic antidepressants.

multilexcode	Product Name
00661003	NORVAL tablets 30mg [BENCARD]
00661001	NORVAL tablets 10mg [BENCARD]
03556010	TRAZODONE capsules 100mg [WINTHROP]
01687001	MOLIPAXIN sugar free liquid 50mg/5ml [AVENTIS]
03577010	TRAZODONE capsules 100mg [TEVA]
03556009	TRAZODONE capsules 50mg [WINTHROP]
01513003	MOLIPAXIN tablets 150mg [AVENTIS]
03704010	TRAZODONE capsules 100mg [GEN (UK)]
04857003	trazodone tablets 150mg
04857002	trazodone capsules 100mg
03577009	TRAZODONE capsules 50mg [TEVA]
01513002	MOLIPAXIN capsules 100mg [AVENTIS]
04857001	trazodone capsules 50mg
03273010	TRAZODONE capsules 100mg [HILLCROSS]
03273009	TRAZODONE capsules 50mg [HILLCROSS]
03704009	TRAZODONE capsules 50mg [GEN (UK)]
04858002	trazodone modified release tablet 150mg
04858001	trazodone sugar free oral solution 50mg/5ml
03704011	TRAZODONE tablets 150mg [GEN (UK)]
01687002	MOLIPAXIN controlled release tablet 150mg [AVENTIS]
03556011	TRAZODONE tablets 150mg [WINTHROP]
03577011	TRAZODONE tablets 150mg [TEVA]
01513001	MOLIPAXIN capsules 50mg [AVENTIS]
01863001	SURMONTIL tablets 10mg [AVENTIS]
04892001	trimipramine maleate tablets 10mg
06159009	TRIMIPRAMINE tablets 25mg [HILLCROSS]
01863002	SURMONTIL tablets 25mg [AVENTIS]
04892002	trimipramine maleate tablets 25mg
04892003	trimipramine maleate capsules 50mg
06158009	TRIMIPRAMINE tablets 10mg [HILLCROSS]

eTable 1: Codes used to defined tricyclic antidepressants.

multilexcode	Product Name
01863003	SURMONTIL capsules 50mg [AVENTIS]
04375001	viloxazine hydrochloride tablets 50mg
01040001	VIVALAN tablets 50mg [ASTRAZENEC]
03557001	desipramine tablets 25mg
01853001	PERTOFRAN tablets 25mg [NOV/GEIGY]
02492001	CONCORDIN 5 tablets [M S D]
02494001	CONCORDIN 10 tablets [M S D]
04627001	protriptyline tablets 5mg
04627002	protriptyline tablets 10mg
01932010	AMITRIPTYLINE sugar free oral solution 50mg/5ml [ROSEMONT]
00134009	AMITRIPTYLINE tablets 25mg [CROSS-PHAR]
00136009	AMITRIPTYLINE tablets 10mg [IVAX]
05923009	AMITRIPTYLINE tablets 25mg [ALMUS]
01870002	TRYPTIZOL MR capsules 75mg [M S D]
01869001	TRYPTIZOL tablets 10mg [M S D]
00130010	AMITRIPTYLINE tablets 50mg [BERK]
02776003	amitriptyline hydrochloride tablets 50mg
00136010	AMITRIPTYLINE tablets 25mg [IVAX]
00128010	AMITRIPTYLINE tablets 25mg [TEVA]
00133009	AMITRIPTYLINE tablets 10mg [ACTAVIS]
00129011	AMITRIPTYLINE tablets 50mg [WOCKHARDT]
01849001	DOMICAL tablets 10mg [BERK]
00138009	AMITRIPTYLINE tablets 10mg [SUSSEX]
01861001	LENTIZOL capsules 25mg [PFIZER]
02819001	ELAVIL tablets 10mg [DDSA]
03074001	amitriptyline hydrochloride modified release capsules 25mg
00131010	AMITRIPTYLINE tablets 25mg [HILLCROSS]
02776001	amitriptyline hydrochloride tablets 10mg
00128009	AMITRIPTYLINE tablets 10mg [TEVA]
00131009	AMITRIPTYLINE tablets 10mg [HILLCROSS]

eTable 1: Codes used to defined tricyclic antidepressants.

multilexcode	Product Name
00137009	AMITRIPTYLINE tablets 25mg [REGENT]
00129009	AMITRIPTYLINE tablets 10mg [WOCKHARDT]
00133010	AMITRIPTYLINE tablets 25mg [ACTAVIS]
00130009	AMITRIPTYLINE tablets 10mg [BERK]
03074002	amitriptyline hydrochloride modified release capsules 50mg
00132010	AMITRIPTYLINE tablets 25mg [CELLTECH]
01932011	AMITRIPTYLINE sugar free oral solution 10mg/5ml [ROSEMONT]
00135009	AMITRIPTYLINE tablets 10mg [KENT]
00131011	AMITRIPTYLINE tablets 50mg [HILLCROSS]
01849002	DOMICAL tablets 25mg [BERK]
07191002	amitriptyline hydrochloride sugar free oral solution 25mg/5ml
01869002	TRYPTIZOL tablets 25mg [M S D]
01869003	TRYPTIZOL tablets 50mg [M S D]
00133011	AMITRIPTYLINE tablets 50mg [ACTAVIS]
01871001	TRYPTIZOL sugar free mixture 10mg/5ml [M S D]
00135010	AMITRIPTYLINE tablets 25mg [KENT]
01861002	LENTIZOL capsules 50mg [PFIZER]
02776002	amitriptyline hydrochloride tablets 25mg
07191003	amitriptyline hydrochloride sugar free oral solution 10mg/5ml
01849003	DOMICAL tablets 50mg [BERK]
07191001	amitriptyline hydrochloride sugar free oral solution 50mg/5ml
00128011	AMITRIPTYLINE tablets 50mg [TEVA]
00129010	AMITRIPTYLINE tablets 25mg [WOCKHARDT]
00135011	AMITRIPTYLINE tablets 50mg [KENT]
01932009	AMITRIPTYLINE sugar free oral solution 25mg/5ml [ROSEMONT]
03074003	amitriptyline hydrochloride modified release capsules 75mg
00130011	AMITRIPTYLINE tablets 25mg [BERK]
00138010	AMITRIPTYLINE tablets 25mg [SUSSEX]
01845001	AVENTYL capsules 10mg [LILLY]
04303002	nortriptyline tablets 25mg

eTable 1: Codes used to defined tricyclic antidepressants.

multilexcode	Product Name
01847001	ALLEGRON tablets 10mg [KING]
04303001	nortriptyline tablets 10mg
01845002	AVENTYL capsules 25mg [LILLY]
01847002	ALLEGRON tablets 25mg [KING]
04304002	nortriptyline capsules 25mg
04304001	nortriptyline capsules 10mg
04304003	nortriptyline liquid 10mg/5ml
01845003	AVENTYL liquid 10mg/5ml [LILLY]
00443010	IMIPRAMINE tablets 25mg [CP PHARM]
01859002	TOFRANIL tablets 25mg [NOVARTIS]
01859003	TOFRANIL syrup 25mg/5ml [NOVARTIS]
01859001	TOFRANIL tablets 10mg [NOVARTIS]
00445009	IMIPRAMINE tablets 10mg [HILLCROSS]
01850009	IMIPRAMINE tablets 25mg [TEVA]
00444009	IMIPRAMINE tablets 10mg [ACTAVIS]
00444010	IMIPRAMINE tablets 25mg [ACTAVIS]
02908001	PRAMINIL tablets 10mg [DDSA]
02887002	imipramine tablets 25mg
02887001	imipramine tablets 10mg
00445010	IMIPRAMINE tablets 25mg [HILLCROSS]
03869001	imipramine syrup 25mg/5ml
05316009	IMIPRAMINE tablets 10mg [TEVA]
17567001	imipramine oral solution 25mg/5ml

eTable 2: Codes used to defined selective serotonin reuptake inhibitor prescriptions (except paroxetine).

multilexcode	Product Name
14029001	CIPRALEX oral drops 10mg/ml [LUNDBECK]
17209001	CIPRALEX oral drops 20mg/ml [LUNDBECK]
11714001	CIPRALEX tablets 10mg [LUNDBECK]
01911001	CIPRALEX tablets 20mg [LUNDBECK]
12337001	CIPRALEX tablets 5mg [LUNDBECK]
07827001	CIPRAMIL oral drops 40mg/ml [LUNDBECK]
08604002	CIPRAMIL tablets 10mg [LUNDBECK]
08604001	CIPRAMIL tablets 20mg [LUNDBECK]
08604003	CIPRAMIL tablets 40mg [LUNDBECK]
07825001	citalopram oral drops 40mg/ml
08619002	citalopram tablets 10mg
05061009	CITALOPRAM tablets 10mg [ACTAVIS]
06051009	CITALOPRAM tablets 10mg [ALMUS]
04664009	CITALOPRAM tablets 10mg [GEN (UK)]
04366009	CITALOPRAM tablets 10mg [HILLCROSS]
06005009	CITALOPRAM tablets 10mg [IVAX]
04331009	CITALOPRAM tablets 10mg [NEOLAB]
04294009	CITALOPRAM tablets 10mg [SANDOZ]
04578009	CITALOPRAM tablets 10mg [STERWIN]
05104009	CITALOPRAM tablets 10mg [TEVA]
08619001	citalopram tablets 20mg
05062009	CITALOPRAM tablets 20mg [ACTAVIS]
04665009	CITALOPRAM tablets 20mg [GEN (UK)]
04367009	CITALOPRAM tablets 20mg [HILLCROSS]
04332009	CITALOPRAM tablets 20mg [NEOLAB]
04729009	CITALOPRAM tablets 20mg [NICHE]
04295009	CITALOPRAM tablets 20mg [SANDOZ]

04579009	CITALOPRAM tablets 20mg [STERWIN]
05105009	CITALOPRAM tablets 20mg [TEVA]

eTable 2: Codes used to defined selective serotonin reuptake inhibitor prescriptions (except paroxetine (cont.)).

08619003	citalopram tablets 40mg
04666009	CITALOPRAM tablets 40mg [GEN (UK)]
04368009	CITALOPRAM tablets 40mg [HILLCROSS]
04296009	CITALOPRAM tablets 40mg [SANDOZ]
14028001	escitalopram oral drops 10mg/ml
17208001	escitalopram oral drops 20mg/ml
08328001	escitalopram tablets 10mg
01438001	escitalopram tablets 20mg
12336001	escitalopram tablets 5mg
03507002	FAVERIN tablets 100mg [SOLVAY]
03507001	FAVERIN tablets 50mg [SOLVAY]
00407001	FELICIUM capsules 20mg [OPUS]
05552001	fluoxetine capsules 20mg
03345009	FLUOXETINE capsules 20mg [GEN (UK)]
03356009	FLUOXETINE capsules 20mg [GENUS]
03270009	FLUOXETINE capsules 20mg [HILLCROSS]
03290009	FLUOXETINE capsules 20mg [IVAX]
06933009	FLUOXETINE capsules 20mg [MILPHARM]
03348009	FLUOXETINE capsules 20mg [NICHE]
03727009	FLUOXETINE capsules 20mg [RANBAXY]
03325009	FLUOXETINE capsules 20mg [TEVA]
04611009	FLUOXETINE capsules 20mg [TILLOMED]
03393009	FLUOXETINE capsules 20mg [WINTHROP]
05552003	fluoxetine capsules 60mg
04389009	FLUOXETINE capsules 60mg [GEN (UK)]
05552002	fluoxetine oral solution 20mg/5ml
04186009	FLUOXETINE oral solution 20mg/5ml [HILLCROSS]
04573009	FLUOXETINE oral solution 20mg/5ml [IVAX]

15596001	fluoxetine sugar free oral solution 20mg/5ml
04179009	FLUOXETINE syrup 20mg/5ml [TEVA]

eTable 2: Codes used to defined selective serotonin reuptake inhibitor prescriptions (except paroxetine (cont.)).

03506002	fluvoxamine tablets 100mg
03506001	fluvoxamine tablets 50mg
06825002	LUSTRAL tablets 100mg [PFIZER]
06825001	LUSTRAL tablets 50mg [PFIZER]
09185001	OXACTIN capsules 20mg [DISCOVERY]
12748001	PAXORAN tablets 10mg [RANBAXY]
12749001	PAXORAN tablets 20mg [RANBAXY]
05509001	PROZAC capsules 20mg [LILLY]
05509003	PROZAC capsules 60mg [LILLY]
05509002	PROZAC liquid 20mg/5ml [LILLY]
15563001	PROZEP oral solution 20mg/5ml [CHEMIDEX]
09233001	PROZIT oral solution 20mg/5ml [PINEWOOD]
09840001	RANFLUTIN capsules 20mg [RANBAXY]
06826002	sertraline tablets 100mg
06826001	sertraline tablets 50mg
06250009	SERTRALINE tablets 50mg [ACTAVIS]
06156009	SERTRALINE tablets 50mg [HILLCROSS]
17632001	fluoxetine tablets 10mg
13840001	sertraline oral suspension 50mg/5ml

eTable 3: Codes used to defined paroxetine prescriptions

multilexcode	Product Name
06509003	paroxetine sugar-free suspension 20mg/10ml
14617001	paroxetine tablets 10mg
06509001	paroxetine tablets 20mg
04421009	PAROXETINE tablets 20mg [ACTAVIS]
04649009	PAROXETINE tablets 20mg [GEN (UK)]
05579009	PAROXETINE tablets 20mg [GENUS]
04667009	PAROXETINE tablets 20mg [HILLCROSS]
03912009	PAROXETINE tablets 20mg [IVAX]
06509002	paroxetine tablets 30mg
04971009	PAROXETINE tablets 30mg [ACTAVIS]
04992009	PAROXETINE tablets 30mg [HILLCROSS]
06510003	SEROXAT sugar-free suspension 20mg/10ml [GLAXSK PHA]
15192001	SEROXAT tablets 10mg [GLAXSK PHA]
06510001	SEROXAT tablets 20mg [GLAXSK PHA]
06510002	SEROXAT tablets 30mg [GLAXSK PHA]

etable 4: Association of actual prescription with physicians' previous prescription, a count of the physicians' previous three prescriptions and indicators for the physicians' previous seven prescriptions, adjusted for year of first prescription. TCAs vs. SSRIs.

	TCAs vs. SSRIs					
Instrument:	One prior prescription Risk difference (95% confidence interval)		Count of three prior prescription Risk difference (95% confidence interval)		Indicators for seven prior prescription Risk difference (95% confidence interval)	
Prior prescription	14.90 (14.42,15.38)					
1 prior prescription			10.90 (10.43,11.37)		9.07 (8.82,9.33)	
2 prior prescriptions			21.35 (20.71,21.98)		7.61 (7.35,7.86)	
3 prior prescriptions			32.60 (31.77,33.44)		7.25 (7.01,7.49)	
4 prior prescriptions					6.71 (6.45,6.96)	
5 prior prescriptions					6.91 (6.66,7.17)	
6 prior prescriptions					6.82 (6.56,7.07)	
7 prior prescriptions					6.68 (6.42,6.94)	
N	886,735		886,735		847,405	
Number of physicians	6,555		6,555		6,555	
Standard error on prescription coefficient	0.14		0.10		0.09	
F-Test	F(1,6554)	3,663	F(3,552)	1,971	F(7,6548)	1,469
Partial r^2	0.02		0.05		0.07	

Notes: SSRI=Selective serotonin reuptake inhibitors, TCA=tricyclic antidepressant. All confidence intervals robust for heteroskedasticity and clustered by physician. Risk difference is difference in probability of tricyclic antidepressant or paroxetine actually being prescribed if physician previously prescribed a paroxetine or tricyclic antidepressant. E.g. In row 1, column 1, if the physician previously prescribed a tricyclic antidepressant, their current patient is 15% more likely to also be prescribed a tricyclic antidepressant than a selective serotonin reuptake inhibitor. There are fewer observations in the final column because some patients' physicians had issued fewer than seven previous SSRI or TCA prescriptions.

etable 5: Association of actual prescription with physicians' previous prescription, a count of the physicians' previous three prescriptions and indicators for the physicians' previous seven prescriptions, adjusted for year of first prescription. Paroxetine vs. SSRIs.

	Paroxetine vs. SSRIs					
Instrument:	One prior prescription Risk difference (95% confidence interval)		Count of three prior prescription Risk difference (95% confidence interval)		Indicators for seven prior prescription Risk difference (95% confidence interval)	
Prior prescription	27.72 (26.69,28.76)					
1 prior prescription			12.67 (12.16,13.18)		15.01 (14.43,15.59)	
2 prior prescriptions			31.77 (30.76,32.78)		11.19 (10.65,11.73)	
3 prior prescriptions			54.58 (53.15,56.02)		10.03 (9.50,10.55)	
4 prior prescriptions					7.67 (7.14,8.21)	
5 prior prescriptions					7.12 (6.61,7.63)	
6 prior prescriptions					6.70 (6.21,7.20)	
7 prior prescriptions					6.53 (6.05,7.02)	
N	390,600		390,600		359,736	
Number of physicians	5,144		5,144		5,144	
Standard error on prescription coef	0.20		0.15		0.14	
F-Test	F(1,5143)	2,770	F(3,5143)	2,582	F(7,5143)	1,429
Partial r^2	0.08		0.13		0.16	

Notes: SSRI=Selective serotonin reuptake inhibitors. All confidence intervals robust for heteroskedasticity and clustered by physician. Risk difference is difference in probability of SSRI or paroxetine actually being prescribed if physician previously prescribed a paroxetine or other SSRI. E.g. In row 1, column 1, if the physician previously prescribed paroxetine, their current patient is 28% more likely to also be prescribed paroxetine than another selective serotonin reuptake inhibitor. There are fewer observations in the final column because some patients' physicians had issued fewer than seven previous SSRI prescriptions.

etable 6: Association of covariates with actual prescription, and physicians' previous prescriptions, a count of the physicians' previous three prescriptions and indicators for the physicians' previous seven prescriptions, adjusted for year of first prescription. TCAs vs. SSRIs vs. (N=886,735)

	Actual prescription TCAs vs.SSRIs F-stat	Physicians' prior prescription F-stat	Count of three previous prescription F-stat	Indicators for physicians' seven previous prescriptions F-stat
Body Mass Index > 25 (N=679,755)	2360.89	45.13	34.05	15.00
Hospitalized in prior year	6.72	0.38	1.49	1.55
Consultations in prior year>13	8423.57	104.19	49.79	12.38
Age at first prescription >40	12106.78	310.92	136.68	48.90
Prescription in prior year>5	14113.39	252.04	112.03	35.75
Male*	122.27	10.29	7.53	5.70
Ever smoked	2741.45	161.24	73.88	27.80
Diagnosed depressed prior to prescription	3758.00	149.73	57.00	23.44
Prior diagnosis definite self-harm	0.55	9.29	3.23	1.82
Prior hypnotic prescription	983.04	36.23	20.83	10.08
Prior anti-psychotic prescription	95.03	1.87	1.38	0.16
Prior Charlson Index not zero	3231.43	79.34	50.53	14.24
Percent prescribed before 2004	169.77	176.17	90.00	NA

Notes: SSRI=Selective serotonin reuptake inhibitors, TCA=tricyclic antidepressant. All F-statistics calculated with robust standard errors clustered by physician. The sample for the seven previous prescriptions was 847,405. The null hypothesis of the F-test is that the coefficient(s) on the prescription or previous prescription was (were jointly) equal to zero.

etable 7: Association of covariates with actual prescription, and physicians' previous prescriptions, a count of the physicians' previous three prescriptions and indicators for the physicians' previous seven prescriptions, adjusted for year of first prescription. Paroxetine vs. SSRIs. (N=390,600)

	Actual prescription Paroxetine vs.<br>F-stat	Physicians' prior prescription<br>F-stat	Count of previous<br>F-stat	Indicators for physicians' seven previous prescriptions<br>F-stat
Body Mass Index > 25 (N=290,301)	7.23	2.88	1.29	0.51
Hospitalized in prior year	0.03	0.63	2.50	1.57
More than 13 Consultations in prior year	40.96	0.62	2.62	0.81
Older than 40 at first prescriptions	0.93	0.04	0.46	0.49
More than 5 prescriptions in prior year	36.11	0.23	0.51	1.00
Male*	129.61	2.61	6.89	1.50
Ever smoked	1.40	0.66	1.00	1.86
Diagnosed depressed prior to prescription	82.80	0.82	7.73	1.02
Prior diagnosis definite self-harm	4.73	3.92	2.45	1.69
Prior hypnotic prescriptions	0.88	1.45	3.11	1.42
Prior anti-psychotic prescriptions	45.91	0.004	5.36	0.12
Prior Charlson Index not zero	17.70	2.17	0.52	1.05
Percent prescribed before 2004	11963.87	11974.89	5141.24	NA

Notes: SSRI=Selective serotonin reuptake inhibitors. All F-statistics calculated with robust standard errors clustered by physician. The sample for the seven previous prescriptions was 359,736. The null hypothesis of the F-test is that the coefficient(s) on the prescription or previous prescription was (were jointly) equal to zero.
